# Investigating Ghanaian *Allium* Species for Anti-Infective and Resistance-Reversal Natural Product Leads to Mitigate Multidrug-Resistance in Tuberculosis

**DOI:** 10.3390/antibiotics10080902

**Published:** 2021-07-23

**Authors:** Cynthia Amaning Danquah, Michael Tetteh, Isaac Kingsley Amponsah, Abraham Yeboah Mensah, Kwame Ohene Buabeng, Simon Gibbons, Sanjib Bhakta

**Affiliations:** 1 Department of Pharmacology, Faculty of Pharmacy and Pharmaceutical Sciences, Kwame Nkrumah University of Science and Technology (KNUST), PMB, Kumasi, Ghana; mitetteh26@gmail.com; 2 Department of Pharmacognosy, Faculty of Pharmacy and Pharmaceutical Sciences, KNUST, PMB, Kumasi, Ghana; akila.amponsah@gmail.com (I.K.A.); aymensah.pharm@knust.edu.gh (A.Y.M.); 3 Department of Pharmacy Practice, Faculty of Pharmacy and Pharmaceutical Sciences, KNUST, PMB, Kumasi, Ghana; kobuabeng.pharm@knust.edu.gh; 4 Norwich Research Park, School of Pharmacy, University of East Anglia, Norwich NR4 7TJ, UK; s.gibbons@uea.ac.uk; 5 Department of Biological Sciences, Institute of Structural and Molecular Biology, Birkbeck, University of London, Malet Street, London WC1E 7HX, UK; s.bhakta@bbk.ac.uk

**Keywords:** tuberculosis, HT-SPOTi, efflux pumps, biofilms, antimicrobial resistance

## Abstract

The bulbs of *Allium* species are a known source of antibacterial phytochemicals. Anti-infective, efflux pump and biofilm inhibitory activities of bulb extracts of selected Ghanaian shallots *Allium cepa* var aggregatum were evaluated using the HT-SPOTi assay and other whole-cell phenotypic screening techniques to determine their possible mechanisms of action. Ethanol and aqueous extracts of white *A. cepa* inhibited the growth of *Mycobacterium smegmatis* mc^2^ 155 and *Escherichia coli,* respectively. The majority of the *Allium* extracts significantly (*p* < 0.05) exhibited efflux pump inhibitory activity against all the acid-fast, Gram-positive and Gram-negative strains used. Hexane and chloroform extract of the pink *A. cepa* and the aqueous extract of the white *A. cepa* significantly inhibited *M. smegmatis* biofilm formation. For *Pseudomonas aeruginosa*, the inhibition was observed at 250 µg/mL for the aqueous extract (~77.34%) and 125 µg/mL for the hexane extract (~76.51%). The results suggest that Ghanaian shallots could potentially be useful when further developed to tackle antimicrobial resistance, particularly in tuberculosis (TB).

## 1. Introduction

A surge in the emergence of multidrug- and extensively-drug-resistant strains of *Mycobacterium tuberculosis*, has been linked to the increasing failure of antimicrobial therapy as a result of irrational use of antibiotics, and non-compliance to conventional treatment. There is, therefore, renewed research interest in natural products with the hope of discovering new antitubercular leads.

Antimicrobial resistance (AMR) has been named by the World Health Organization (WHO) as one of the three most important public health threats of the 21st century [1], and it causes persistent infections that claim millions of lives every year with enormous demands on medical and social resources. A WHO Interagency Coordination Group report on AMR estimates that 700,000 people die each year due to antibiotic resistance including 230,000 people who die from multidrug-resistant tuberculosis. This number is projected to rise to 10 million by 2050 if nothing is done to stop the AMR menace [2,3]. The overuse and misuse of antimicrobial medications, arbitrary prescribing, and unnecessary use of antibiotics in agriculture are just some of the causes of AMR. Diminishing numbers of new antibiotics in the drug development pipeline has intensified the urgency to develop new antimicrobials with novel modes of action or resistance-reversal mechanisms for the treatment of infections.

Biofilms and efflux pumps have been identified as key contributors to AMR. Biofilm-producing cells are known to be much less susceptible to antimicrobial agents than their planktonic forms, rendering them to be 1000-fold more resistant to antibacterial agents. Bacterial efflux pumps are membrane proteins involved in the export of antibiotics from the bacterial cell, making infections more difficult to treat [4]. Over-expression of efflux proteins in *Mycobacterium* species including *M. tuberculosis* plays an important role in antibiotic resistance and the development of multidrug resistance (MDR) phenotypes [5,6].

Ojah et al. (2008) [7] hypothesized that biofilm formation in *M. tuberculosis* could also be responsible for persistence of *Mycobacterium* infections. In the same work, *M. tuberculosis* and *M. smegmatis* biofilms were able to tolerate ten times the minimal inhibitory concentrations of the anti-tuberculosis drugs isoniazid and rifampicin [7]. The simultaneous use of efflux pump and/or biofilm inhibitors would target bacterial growth and AMR. This would therefore provide a potential way to improve the treatment of MDR infections.

A significant percentage of newly approved antibacterials are either natural products or their derivatives [8]. Additionally, hundreds of natural products possessing novel and known structural architectures have been reported to exhibit activity towards non-resistant and multidrug-resistant strains of *M*. *tuberculosis*. Recent research has reported the activity of plant natural products that inhibit biofilm formation and efflux pumps in bacteria [9,10]. Ethnopharmacological studies have shown that plants from the Amaryllidaceae family such as garlic, onions and bulbs of harmattan lily (*Crinum jagus*) are being used in Ghana for various respiratory tract conditions such as cough, asthma and even tuberculosis. This includes *Allium sativum* (bulb) and *Allium cepa* (bulb and leaves) [11,12]. Extracts and isolated compounds from this genus have also shown promising effects as inhibitors of efflux pumps and bacterial biofilms [13,14]. In 2018 we reported on the efflux pump inhibition and anti-biofilm activity of disulfides from *Allium stipitatum* and their synthetic derivatives on mycobacterial biofilms and efflux pumps [14].

**Figure 1:**
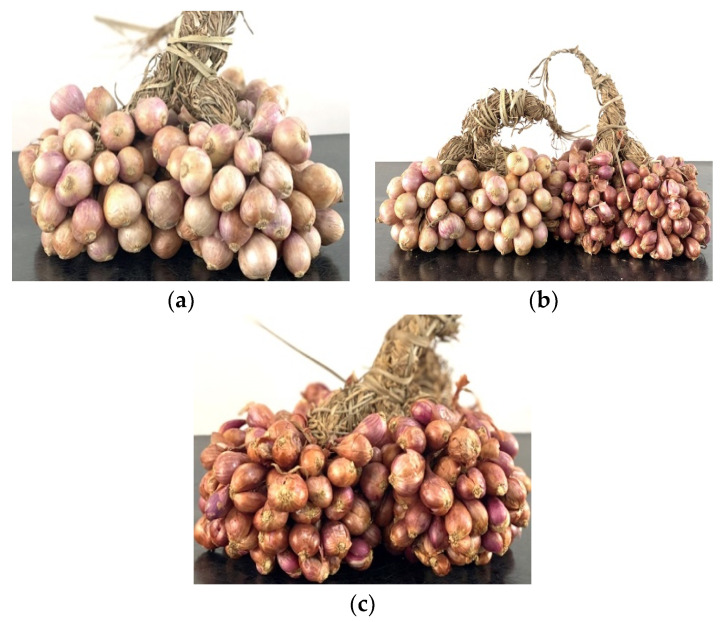
Photos of *Allium cepa var aggregatum* (White) (**a**); *Allium cepa var aggregatum* (White and Pink varieties together) (**b**) and *Allium cepa var aggregatum* (Pink) (**c**).

In this study, we investigated the antibacterial, efflux pump and biofilm formation inhibitory activities of the bulb extracts of Ghanaian *Allium cepa* var *aggregatum* pink and white varieties (shallot) as potentially useful leads in our efforts towards the discovery and development of new anti-tuberculosis agents. Photos of *Allium cepa* var *aggregatum* white variety (a), white and pink varieties together (b) and the pink variety (c) are shown in Figure 1 respectively.

## 2. Results

### 2.1. Phytochemical Screening for the Allium Extracts

Initial phytochemical screening confirmed the presence of various secondary metabolites including condensed tannins, alkaloids, triterpenoids, phytosterols and coumarins in majority of the extracts as shown in Table 1.

### 2.2. Allium Extracts Demonstrated Antibacterial Growth Inhibition

The antibacterial activity of the *Allium* extracts was evaluated by determining the minimum inhibitory concentration (MIC) (Table 2). Extracts were moderately effective at different concentrations against the pathogens used (MIC values ranged between 250–500 µg/mL). *Escherichia coli* was sensitive to the methanol extract of pink *A. cepa*, methanol and aqueous extract of white *A. cepa* with the aqueous extract of the white *A. cepa* having the highest inhibitory effect (MIC-250 µg/mL). At a concentration of 500 µg/mL, the methanol extract of pink *A. cepa* and ethanol and aqueous extract of white *A. cepa* inhibited the growth of *P. mirabilis*. Methanol extracts of pink *A. cepa* at 250 µg/mL and ethanol and hexane extracts at 500 µg/mL inhibited *S. aureus*. For *K. pneumoniae* and *S. typhi*, which were not sensitive to amoxicillin (MIC->500 µg/mL), only the ethanol extract of pink *A. cepa* was effective: *K. pneumoniae* (MIC-500 µg/mL) while the white *A. cepa* aqueous extract inhibited *S. typhi* (MIC-500 µg/mL). The *P. aeruginosa* was sensitive to all the extracts with the exception of the aqueous extract of the white *A. cepa*. The methanol extract of the pink *A. cepa* gave the highest activity at a concentration of 250 µg/mL with the other extracts at a concentration of 500 µg/mL. In the case of *S. epidermidis*, the ethanol, methanol and chloroform extract of pink *A. cepa* and the methanol extract of white *A. cepa* had an inhibitory effect at 500 µg/mL. The *Mycobacterium* strain *M. smegmatis* was sensitive to the ethanol extract of the pink *A. cepa* at a concentration of 250 µg/mL. However, the rest of the extracts showed no activity.

### 2.3. Allium Extracts Demonstrate Efflux Pump Inhibitory Effect

**Figure 2:**
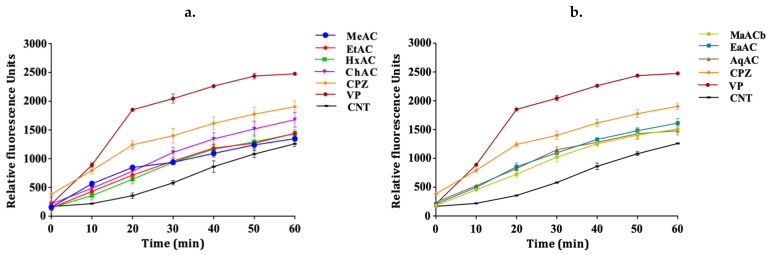
Effect of *A. cepa* extracts on the accumulation of EtBr in *M. smegmatis.* (**a**) *A. cepa* pink: MeAC-methanol, EtAC-ethanol, HxAC-hexane and ChAC-chloroform; (**b**) *Allium cepa* white: MeACb-methanol, EaAC-ethanol, AqAC-aqueous, CPZ-chlorpromazine; VP-verapamil; CNT-control.

**Figure 3:**
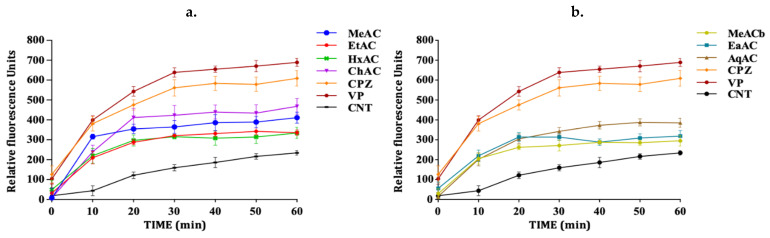
Effect of *A. cepa* on the accumulation of EtBr in *P. aeruginosa.* (**a**) *A. cepa* pink: MeAC-methanol, EtAC-ethanol, HxAC-hexane and ChAC-chloroform; (**b**) *Allium cepa* white: MeACb-methanol, EaAC-ethanol, AqAC-aqueous, CPZ—chlorpromazine; VP-verapamil; CNT-control.

**Figure 4:**
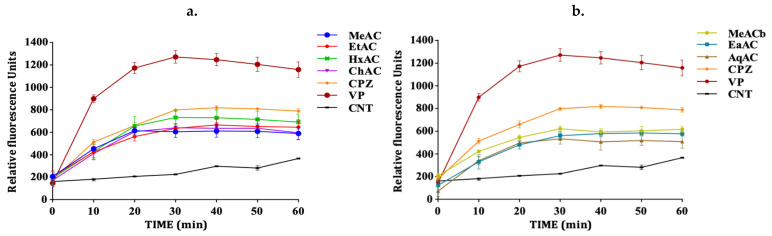
Effect of *A. cepa* on the accumulation of EtBr in *S. aureus.* (**a**) *A. cepa* pink: MeAC-methanol, EtAC-ethanol, HxAC-hexane and ChAC-chloroform; (**b**) *Allium cepa* white: MeACb-methanol, EaAC-ethanol, AqAC-aqueous, CPZ—chlorpromazine; VP-verapamil; CNT-control.

The efflux pump inhibition assay was used to evaluate the activity of the extracts using ethidium bromide (EtBr) as a substrate. This assay is based on the principle that EtBr has a strong fluorescence property intracellularly. It binds to DNA to give an increasing fluorescence following continual accumulation. When accumulated within the bacterial cells, fluorescence intensity increases, giving an indication of the inhibition of the efflux pump mechanism within the cells [14]. In the absence of an efflux pump inhibitory activity, the cell pumps out EtBr, resulting in a low fluorescence measurement. The accumulation assay was performed with sub-MIC concentration of the extracts (½ MIC). The results revealed that the extracts significantly (*p*-value < 0.05) enhanced the accumulation of EtBr to different levels when compared to the control with no efflux pump inhibition. This confirms their inhibitory activity against bacterial efflux pumps. The methanolic extract of the pink *A. cepa* was the only extract that showed no significant activity in the *M. smegmatis* strain (Figure 2a) (*p*-value > 0.05). However, all the extracts of white *A*. *cepa* significantly enhanced the accumulation of EtBr (Figure 2b). All extracts significantly enhanced EtBr accumulation in *P. aeruginosa* (Figure 3). The effect of efflux pump inhibition in *S*. *aureus* (Figure 4a) was statistically significant for all the extracts of the pink *A. cepa*. Extracts of the white *A. cepa* also showed a significant inhibitory effect, with the exception of the methanol (Figure 4b). The chloroform extract of the pink *A. cepa* had a greater efflux pump inhibitory activity in the *M. smegmatis* and *P. aeruginosa* strain than the other plant extracts. The hexane extract of the pink variety of *A. cepa* is seen to have the strongest activity among the extracts in the *S. aureus* strain (Figure 4a). Its effect is statistically (*p*-value < 0.05) comparable to that of chlorpromazine, a known efflux inhibitor in *S. aureus*.

The standard efflux pump inhibitor verapamil exhibited a very good efflux pump inhibitory activity in all the bacterial cells followed by chlorpromazine.

### 2.4. Biofilm Formation

**Figure 5:**
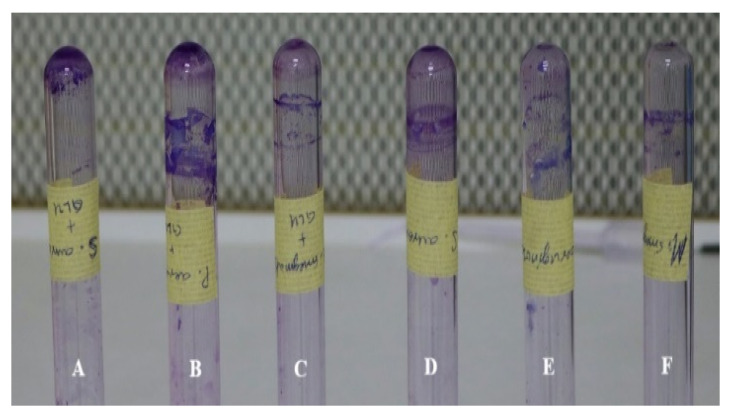
Biofilm formation by (**A**) *S. aureus* + glucose; (**B**) *P. aeruginosa* + glucose; (**C**) *M. smegmatis* + glucose; (**D**) *S. aureus*; (**E**) *P. aeruginosa;* (**F**) *M. smegmatis*.

The result from the crystal violet staining assay showed that biofilm formation was high in the TSB medium supplemented with additional glucose to 1% than the medium without glucose supplement (Figure 5). Similar results were observed in the microtitre plate method (results not shown). For better biofilm formation we recommend that TSB be supplemented with additional glucose.

### 2.5. Allium Extracts Demonstrate Biofilm Inhibition

**Figure 6:**
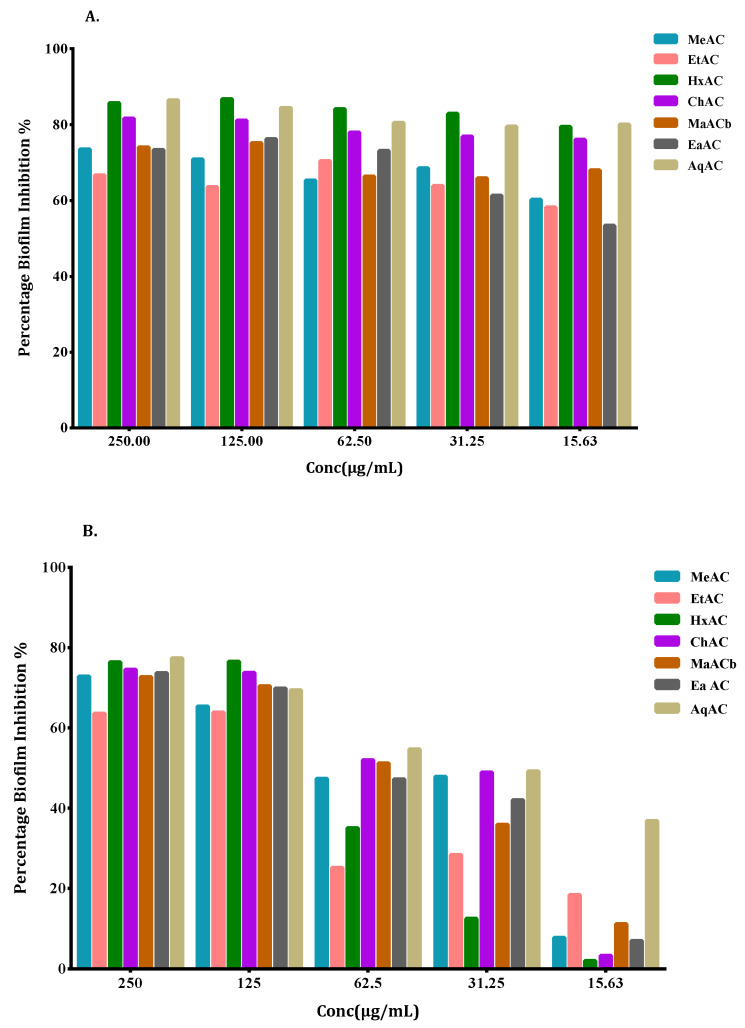

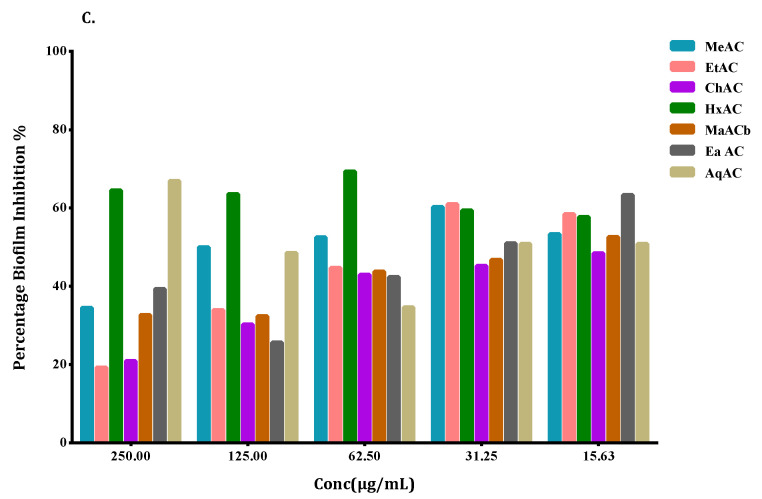
Effects of *A.* *cepa* extracts on biofilm formation in (**A**) *M. smegmatis* (**B**) *P. aeruginosa* (**C**) *S. aureus* strains expressed as percentage (%) inhibition and evaluated by the crystal violet staining assay. Values are the average of at least three independent determinations. Extracts of *A. cepa* pink: MeAC-methanol, EtAC-ethanol, HxAC-hexane and ChAC-chloroform; *Allium cepa* white: MeACb-methanol, EaAC-ethanol, AqAC-aqueous, CPZ-chlorpromazine; VP-verapamil; CNT-control.

Biofilm formation contributes to bacterial persistence, which contributes to anti-microbial resistance. Hence, the *A.* *cepa* extracts were evaluated to find out if they have a biofilm inhibitory effect. The different *Allium* extracts showed varying levels of biofilm inhibition at different concentrations. The biofilm inhibition of the extracts on the *P. aeruginosa* and *M. smegmatis* biofilms were concentration-dependent. A good inhibition of biofilm formation was observed for *M. smegmatis.* Compared to the other extracts, the hexane and chloroform extract of the pink *A. cepa* and the aqueous extract of the white *A. cepa* had significant inhibition on the *M. smegmatis* biofilm (Figure 6A). The hexane extract gave the highest inhibitory effect in all the concentrations used. The aqueous extract of the white *A. cepa* and the hexane extract of the pink *A. cepa* had a greater inhibitory effect in *P. aeruginosa* biofilm formation (Figure 6B). The highest inhibition was observed at 250 µg/mL for the aqueous extract (~77.34%) and 125 µg/mL for the hexane extract (~76.51%).

All the other extracts moderately inhibited biofilm formation in *P*. *aeruginosa* at concentrations of 125 µg/mL and 250 µg/mL. Unlike the *M. smegmatis* and *P. aeruginosa* strains, the *S*. *aureus* strain was less susceptible to biofilm inhibition by the extracts. However, the hexane extract of pink *A. cepa* was significantly effective at inhibiting *S. aureus* biofilms (~69.3%). The aqueous extract of white *A. cepa* was also effective at inhibiting biofilm formation at a sub-inhibitory concentration of 62.5 µg/mL (~66.8%) (Figure 6C).

## 3. Discussion

The minimum inhibitory concentration determined from the HT-SPOTi assay showed that extracts of both white and pink *A. cepa* had a broad spectrum of activity on the Gram-positive and Gram-negative strains at different concentrations. Methanolic and ethanolic extracts of the pink *A. cepa* and the aqueous extract of the white *A. cepa* showed high activity on most of the pathogens. Overall, *P. aeruginosa* was more sensitive to all the extracts with the exception of the aqueous extract of the white *A. cepa*. The methanol extract of the pink *A. cepa* gave the highest activity (MIC-250 µg/mL) while the other extracts (MIC-500 µg/mL). The aqueous extract was the most effective among the white *A. cepa*, inhibiting four pathogens, MIC-250 µg/mL for *E. coli* and 500 µg/mL for *P. mirabilis, P. aeruginosa* and *S. typhi,* respectively. Ethanolic extract of white *A. cepa* had no activity on the pathogens except for *P. mirabilis* (500 µg/mL). In as much as *K. pneumoniae* and *P. mirabilis* are intrinsically insensitive to antibiotics, it is worth noting that some extracts inhibited their growth at 500 µg/mL. Only the ethanol extract of the white *A. cepa* inhibited the growth of *M. smegmatis* with an MIC of 250 µg/mL; even though the activity is moderate, this highlights the need to further explore the extract as a potential antimycobacterial agent since the treatment of mycobacterial infections (notably *M. tuberculosis*) is being threatened by multidrug-resistant strains [6].

In the bacterial efflux pump inhibition assay, the majority of the *Allium* extracts significantly enhanced the accumulation of ethidium bromide in all the strains when compared to the control with no drug. The results suggest that *A. cepa* extracts possess an efflux pump inhibitory activity, hence interfering with ethidium bromide efflux from the bacteria cells that express multiple efflux proteins. This indicates their potential as efflux pump inhibitors. Similar results of efflux pump inhibition by *Allium* plants have been reported; Extracts of *A. sativum* and allyl sulfide (a bioactive component of most *Allium* plants) have been shown to inhibit the multidrug efflux pump EmrD-3 of *V. cholerae* [13]. Disulfides from *A. stipitatum* have also been found to have efflux pump inhibitory activity against *Mycobacterium strains* [14]. Interestingly, some of the *Allium* extracts, which did not show antimicrobial activity (MIC > 500 µg/mL) especially in *M. smegmatis*, were able to inhibit efflux pump activity. These further support the claims that plant extracts are excellent candidates for lead optimization of efflux pump inhibitors [15]. In general, it was observed that the less polar solvent extracts had good activity than the more polar extracts. Drug efflux by efflux pumps is a contributing factor in the growing problem of antimicrobial resistance contributing to the intrinsic and acquired resistance in a wide variety of bacteria. This keeps complicating the treatment of infectious diseases [16,17]. Therefore, the Ghanaian *Allium cepa* var *aggregatum* plant demonstrating to have efflux pump inhibitory properties could have the potential of restoring the bacterial susceptibility to drugs and enhance the treatment of infectious diseases. Further work is underway to isolate and identify the bioactive compounds and also ascertain the modulatory effect of these extracts on drugs in clinical use.

Bacterial biofilms have contributed greatly to the persistence of antimicrobial resistance. They have also become a major leading cause of multi-drug resistance. In the present report, the different extracts of white and pink *A. cepa* varieties inhibited Gram-positive, Gram-negative and *Mycobacterium* biofilms at sub-lethal concentrations. The ability of each extract to inhibit biofilm formation was dependent on the type of solvent and the concentration of the extract used. Overall, non-polar hexane extract of the pink variety of *A. cepa* and the aqueous extract of the white variety inhibited biofilm formation in all strains of bacteria. The biofilm inhibition activity of the extracts against *P. aeruginosa* and *M. smegmatis* were dose-dependent. The underlying mechanisms of the antibiofilm properties of these extracts could be due to, inhibiting the formation of the polymer matrix, suppressing cell adhesion and attachment, interrupting extracellular matrix generation and/or decreasing virulence factors production. These mechanisms have accounted for biofilm inhibition properties of natural products according to Lu et al. in 2019 [18]. In recent years, the antibiofilm properties of *Allium* species have attracted attention. For example, the biofilm inhibition properties of *A. sativum, A. stipitatum*, *A*. *orientale* and *A. porrum* have been documented [19,20,21,22]. The inhibition of bacterial efflux pumps has also been suggested as a strategy to inhibit biofilm formation [23].

It is, therefore, noteworthy that the Ghanaian *A. cepa* var *aggregatum* extracts have demonstrated both efflux pump and biofilm inhibitory properties which are interesting since a major challenge in the management and emergence of multidrug-resistant tuberculosis is the long duration of treatment. Expression of efflux proteins and biofilm formation has been recognized as a common mechanism contributing to both virulence and drug tolerance in *M. tuberculosis* [24]. Richards and Ojha (2014) [25] reported that *Mycobacterium* biofilms enhance the spontaneous growth of mycobacteria, particularly *M. tuberculosis,* and also promotes persistence against exogenous threats. This confers drug resistance to drug-susceptible *M. tuberculosis* mutants [25].

The ability of the extracts to inhibit efflux pumps and biofilm formation in *M. smegmatis* indicates their potential to mitigate *M. tuberculosis* by their pleiotropic mode of action or by their ability to possibly reverse resistance by improving sensitivity when combined with existing antibacterials that have lost their sensitivity due to antibiotic resistance.

## 4. Materials and Methods

### 4.1. Materials

Chlorpromazine, verapamil, rifampicin, ciprofloxacin, EtBr and phosphate-buffered saline (PBS) tablets (0.01 M phosphate buffer, 0.0027 M potassium chloride and 0.137 M sodium chloride, pH 7.4) were purchased from Sigma-Aldrich, St Louis, MO, USA. Middlebrook 7H10 agar, Middlebrook 7H9 broth, oleic acid-albumin-dextrose-catalase (OADC) supplement and albumin–dextrose–catalase (ADC) supplement were purchased from Difco, Franklin Lakes, NJ, USA. Tryptic soy broth (TSB) (HiMedia, Mumbai, India) was used for the anti-biofilm assay. All solutions were prepared in distilled water or DMSO on the day of the experiment.

### 4.2. Collection of Plant Material and Extraction

The pink and white varieties of the bulbs of Ghanaian shallot (*Allium cepa* var *aggregatum*) were purchased from Keta in the Volta region of Ghana (5°55′19.5384″ N 0°59′31.8948″ E) on 13 April 2019. Dr. George Henry Sam, Department of Herbal Medicine, KNUST, Ghana authenticated the plant materials, and voucher specimens (Pink *Allium cepa* var *aggregatum*-KNUST/2019/HM1/013; White *Allium cepa* var *aggregatum*-KNUST/2019/HM1/014) were kept in the Herbarium. The fresh bulbs were washed thoroughly and blended with an Akai (Tokyo, Japan) electric blender (Model no. BD033A SO4M) using hexane, chloroform, ethanol, methanol and water as solvents. The extracts were filtered, and the solvents evaporated using a rotary evaporator (Buchi Rotavapor R-210, BÜCHI Labortechnik AG CH-9230 Flawil 1, Switzerland) to yield semi-solid dark brown extracts. All the extracts were stored in a fridge at −4 °C until used.

### 4.3. Microbial Strains and Culture Conditions

The following microbial strains were used in this study: *Escherichia coli* (ATCC 25922), *Staphylococcus aureus* (ATCC 25923), *Pseudomonas aeruginosa* (ATCC 4853); clinical strains of *Salmonella typhi*, *Klebsiella pneumoniae*, *Staphylococcus epidermidis*, *Proteus mirabilis*; and *Mycobacterium smegmatis* mc^2^ 155 (ATCC 19420), a fast-growing mycobacteria used as a surrogate for *M. tuberculosis*. The pathogens were obtained from the Department of Pharmaceutical Microbiology, and the Cell Culture Laboratory, Department of Pharmacology, KNUST. Glycerol stocks of the microorganisms were stored at −80 °C. Nutrient agar (Oxoid, Basingstoke, England) was used to culture the Gram-positive and Gram-negative bacteria and fungus while Middlebrook 7H10 agar supplemented with OADC was used for *M*. *smegmatis*.

### 4.4. Methods

#### 4.4.1. Antimicrobial Susceptibility Assay with HT-SPOTi

The minimum inhibitory concentrations (MIC) of the different extracts of *cepa* varieties were determined using the high-throughput spot culture growth inhibition (HT-SPOTi) assay as described by Danquah et al. (2016) [26]. Two-fold serial dilutions of a stock concentration of the extracts (50 mg/mL) were done in a PCR half-skirted 96-well plate to give a concentration range of 50–0.05 mg/mL. A volume (2 µL) of the drug dilution was transferred into a corresponding 96-well plate and 200 µL of molten agar was dispensed into it with shaking (nutrient agar was used for the bacteria and fungus, and Middlebrook 7H10 agar with 0.5% (*v*/*v*) glycerol supplemented with 10% OADC for the *Mycobacterium* strain). The plate was left to solidify. A volume of 2 µL of the bacterial suspension containing ~1 × 10^6^ cfu/mL was spotted onto each well of the 96-well plate. The bacterial spot suspension was absorbed into the agar (approximately 5 min). The plate was sealed with parafilm, wrapped with aluminum foil and incubated at 37 °C for 18–48 h based on the doubling time of the bacterial models used in this study. Wells with no drug were added to serve as growth control. Ciprofloxacin, a broad-spectrum antibiotic of the fluoroquinolone class, which is bactericidal and inhibits topoisomerase II (DNA gyrase) and amoxicillin, a broad-spectrum penicillin which is bactericidal and inhibits bacterial cell wall synthesis-were used as drug control for Gram-positive and Gram-negative bacteria. Rifampicin, a first-line TB drug was used for the acid-fast bacteria, *Mycobacterium*. The minimum inhibitory concentration (MIC) was defined as the lowest concentration of antimicrobial agent that inhibited visible bacterial growth within the incubation period. The test was done in triplicate.

#### 4.4.2. Efflux Pump Inhibitory Assay

The whole-cell phenotypic efflux pump inhibition assay was performed according to previously published protocols with some modification using *M. smegmatis, S. aureus* and *P. aeruginosa* [27,28,29,30]. The Gram-positive and Gram-negative cells were cultured in 10 mL nutrient broth while the *Mycobacterium* was cultured in Middlebrook 7H9 broth (Difco, USA) in Erlenmeyer flasks containing 10% OADC enrichment (Becton Dickinson, Franklin Lakes, NJ, USA) and 0.05% Tween 80. They were cultured at 37 °C with shaking at 150 rpm until an optical density of 600 nm (OD_600_) of 0.8–1 (mid-log phase). The OD_600_ of the cultures was adjusted to 0.4 after which 10 mL was centrifuged at 3000 rpm for 10 min. The supernatant was discarded, and pellets washed with sterile PBS.

The pellets were re-suspended in 10 mL of sterile PBS. The bacteria suspension (500 µL) (Test) and 500 µL of PBS (Blank) were pipetted into 2 mL Eppendorf tubes and glucose added to a final concentration of 0.4%. Different concentrations of the extracts and compounds were added at half their MIC’s. The suspension was vortexed to distribute the cells uniformly. Aliquots of 100 µL from each mixture were transferred into 96-well plates (in triplicates) and 5 µL of EtBr added to give a final concentration of 0.5 mg/L. The EtBr was added just before reading the fluorescence intensity. The fluorescence was measured using the microplate reader (Biotek Synergy H1 Hybrid Multi-Mode Reader: 271230, Vermont, VT, USA) at 530 nm (excitation) and 585 nm (emission) every 10 min over a period of 60 min at 37 °C. This measures the effect of the extracts on the accumulation of EtBr within the cells by inhibiting the efflux pumps. The known efflux pump inhibitors, verapamil and chlorpromazine, served as reference drugs and a drug-free culture was used as control.

#### 4.4.3. Biofilm Inhibition Assay

##### Evaluation of Biofilm Forming Potential

The biofilm-forming ability of *M. smegmatis*, *P. aeruginosa* and *S. aureus* was evaluated by the tube method [31] and the microtitre-plate method [32] with minor modifications described below.

##### Tube Method

The tube method is a qualitative assay for the detection of microorganisms that produce biofilm. It is based on the occurrence of a visible film adhering to the surface of the tube. As described by Christensen et al. in 1985 [31], bacterial isolates were inoculated in a polystyrene test tube that contained 5 mL of TSB and incubated at 37 °C for 24 h. 

Cultures supplemented with glucose to a concentration of 1% was included for comparison. The contents were decanted, the tubes washed twice with phosphate-buffered saline and left to dry. Subsequently, the polystyrene tubes were stained with crystal violet (0.1%) for 15 min, rinsed with PBS and air-dried in an inverted position. The occurrence of visible film lined the walls, and the bottom of the tube indicates biofilm production [31]. The tests were carried out in triplicate.

##### Microtitre-Plate Method

The biofilm-forming potential of the microorganisms was quantitatively analyzed using the microtitre-plate biofilm formation assay as previously described with some modifications [32,33,34]. Briefly, a loopful of an overnight culture of the microorganisms were inoculated in 5 mL Tryptone Soy Broth (TSB) and incubated at 37 °C for 24 h with shaking at 150 rpm. The cultures were 1:100 diluted in TSB (supplemented with additional glucose to a final concentration of 1%) after incubation, and 100 µL of each diluted culture pipetted into a 96-well flat-bottom microtitre plate (Star Lab, Humberg, Germany). Plates were covered and incubated at 37 °C. After 24 h, the planktonic cells were aspirated, and wells were washed with PBS to remove planktonic bacteria.

Adherent bacterial biofilms were fixed by drying in the incubator for 30 min and stained with 125 μL of 0.1% (*w*/*v*) crystal violet (Burgoyne Burbidges and Co, Mumbai, India) for 10 min at room temperature. Excess stain was washed with distilled water and left to air dry. Subsequently, the stain was solubilized with 125 µL of 95% ethanol and left for 10 min. The optical density of each well was measured at 600 nm (OD_600_) using an automated plate reader (Biotek Synergy H1 Hybrid Multi-Mode Reader: 271230).

##### Inhibition of Biofilm Formation

The ability of the *Allium* extracts to inhibit biofilm formation was measured using the microtiter plate-based assay as described. A volume (180 µL) of each diluted culture was pipetted into a 96-well flat-bottom microtitre plate. Two-fold serial dilutions of the extracts were made with Tryptic soy broth (TSB) to achieve sub-minimum inhibitory concentrations ranging from 250–15.63 µg/mL. An amount (20 μL) of the extract’s solution was then pipetted into each of the 96-well plates. The plates were incubated undisturbed for 24 h at 37 °C. Wells containing only microorganisms were included to serve as the growth control. After 24 h of incubation at 37 °C, the supernatant was collected and the plates were washed, fixed and stained as described above. The optical density of each well was measured at 600 nm (OD_600_) using an automated plate reader. The bioassay was performed in duplicate. The mean absorbance of the samples was determined, and the percentage inhibition of biofilm was calculated using the equation:

Percentage
Biofilminhibition(%)=(ODnegativecontrol−ODExperimentalODnegativecontrol)×100

### 4.5. Statistical Analysis

GraphPad prism 8.0 software was used for the statistical analysis using One-Way Analysis of Variance (ANOVA). Results were quantified as mean ± SEM. Statistical differences between mean values were performed with Dunnett’s Multiple Comparison Test at *p* < 0.05.

## 5. Conclusions

The findings from this study showed that the bulbs of Ghanaian *Allium cepa* var *aggregatum* (shallots) are a potential source of phytochemical hits to tackle antimicrobial resistance as a result of their inhibition of whole-cell efflux pumps, biofilm formation and antibacterial selectivity against *M. smegmatis* resistance mechanisms. Bioactivity-guided isolation and characterization of compounds from these species could lead to the discovery of lead bioactive compounds that can be developed as drugs for clinical use.

## Figures and Tables

**Table 1:** Phytochemical screening.

	MeAC	HxAC	ChAC	EtAC	AqAC	MeACb	EaAC
Tannins	Condensed	Condensed	Condensed	Condensed	Condensed	Condensed	Condensed
Saponins	+	−	+	+	+	+	+
Flavonoids	+	−	−	+	−	+	−
Reducing sugars	+	−	+	+	+	+	+
Alkaloids	+	+	+	+	+	+−	−
Triterpenoids	+	+	+	+	+	+	+
Phytosterols	+	+	+	+	+	+	+
Coumarins	+	+	+	+	+	+	+

Key: + Detected. − Not detected. *Allium cepa* var *aggregatum* extracts (Pink: MeAC-methanol, HxAC-hexane, ChAC-chloroform, EtAC-ethanol; White: AqAC-aqueous, MeACb-methanol, EaAC-ethanol).

**Table 2:** Effect of extracts of *Allium cepa* on the growth of microorganisms.

	Minimum Inhibitory Concentration MIC (µg/mL)							
	*E. coli*	*P. mirabilis*	*S. aureus*	*K. pneumoniae*	*P. aeruginosa*	*S. typhi*	*S. epidermidis*	*M. smegmatis*
MeAC	500	500	250	>500	250	>500	500	>500
EtAC	>500	>500	500	500	500	>500	500	250
HxAC	>500	>500	500	>500	500	>500	>500	>500
ChAC	>500	>500	>500	>500	500	>500	500	>500
MeACb	500	>500	>500	>500	500	>500	500	>500
EaAC	>500	500	>500	>500	>500	>500	500	>500
AqAC	250	500	>500	>500	500	500	500	>500
Ciprofloxacin	<0.49	<0.49	<0.49	<0.49	<0.49	7.81	<0.49	<0.49
Amoxicillin	15.6	62.5	62.5	>500	62.5	>500	7.81	NT
Rifampicin	NT	NT	NT	NT	NT	NT	NT	7.81

Key: NT = Not tested. *Allium cepa* var *aggregatum* extracts (Pink: MeAC-methanol, HxAC-hexane, ChAC-chloroform, EtAC-ethanol; White: AqAC-aqueous, MeACb-methanol, EaAC-ethanol). Extracts showing activity.

## Data Availability

The data used to support the findings of the study can be made available upon request through the corresponding author.
